# Rate of benign histology after resection of suspected renal cell carcinoma: multicenter comparison between Korea and the United States

**DOI:** 10.1186/s12885-024-11941-3

**Published:** 2024-02-15

**Authors:** Chang Wook Jeong, Jang Hee Han, Seok Soo Byun, Cheryn Song, Sung-Hoo Hong, Jinsoo Chung, Seong Il Seo, Hong Koo Ha, Eu Chang Hwang, Ill Young Seo, Joseph G. Cheaib, Phillip M. Pierorazio, Misop Han, Cheol Kwak

**Affiliations:** 1 Department of Urology, Seoul National University Hospital, 101 Daehak-ro, Jongno-gu, 03080 Seoul, Korea; 2 Department of Urology, Seoul National University Bundang Hospital, Seongnam, Korea; 3 Department of Urology, Asan Medical Center, University of Ulsan College of Medicine, Seoul, Korea; 4 Department of Urology, Seoul St. Mary’s Hospital, College of Medicine, The Catholic University of Korea, Seoul, Korea; 5 Department of Urology, National Cancer Center, Goyang, Korea; 6 Department of Urology, Samsung Medical Center, Sungkyunkwan University School of Medicine, Seoul, Korea; 7 Department of Urology, Pusan National University Hospital, Busan, Korea; 8 Department of Urology, Chonnam National University Hwasun Hospital, Hwasun, Korea; 9 Department of Urology, Institute of Wonkwang Medical Science, Wonkwang University School of Medicine, Iksan, Korea; 10 Department of Urology, Johns Hopkins University School of Medicine, Baltimore, MD USA; 11 Department of Urology, University of Pennsylvania Perelman School of Medicine, Philadelphia, PA USA

**Keywords:** Renal cell carcinoma, Nephrectomy, Benign histology, Diagnostic errors

## Abstract

**Background:**

In the United States, the rate of benign histology among resected renal tumors suspected to be malignant is increasing. We evaluated the rates in the Republic of Korea and assessed the racial effect using recent multi-institutional Korean-United States data.

**Methods:**

We conducted a multi-institutional retrospective study of 11,529 patients (8,812 from The Republic of Korea and 2,717 from the United States) and compared the rates of benign histology between the two countries. To evaluate the racial effect, we divided the patients into Korean, Asian in the US, and Non-Asian in the US.

**Results:**

The rates of benign histology and small renal masses in Korean patients were significantly lower than that in United States patients (6.3% vs. 14.3%, *p* < 0.001) and (≤ 4 cm, 7.6% vs. 19.5%, *p* < 0.001), respectively. Women, incidentaloma, partial nephrectomy, minimally invasive surgery, and recent surgery were associated with a higher rate of benign histology than others.

**Conclusions:**

In Korea, the rate of benign histology among resected renal tumors was significantly lower than that in the United States. This disparity could be caused by environmental or cultural differences rather than racial differences. Our findings suggest that re-evaluating current context-specific standards of care is necessary to avoid overtreatment.

**Supplementary Information:**

The online version contains supplementary material available at 10.1186/s12885-024-11941-3.

## Background

A recent systematic review has demonstrated substantial rates of benign histology (13.3%) in the United States (US) among resected renal tumors suspected to be malignant on preoperative imaging [1]. Furthermore, the estimated number of resected benign renal masses in the US increased by 82% between 2000 and 2009, from 3,098 to 5,624 [1]. Overtreatment is a serious medical issue that should be addressed with caution. The socioeconomic burden should be reduced, as the incidence of misdiagnosed small renal masses (SRMs) is increasing worldwide. However, the current guidelines do not strongly support any clinical prudence [2–4]. Thus, the current standard of care should be critically re-evaluated.

Interestingly, the Korean experience is remarkably different, with lower rates of benign histology [5]. The rate of benign histology was 8.3%, and it was significantly higher among tumors ≤ 4 cm (13.2%) than those > 4 cm (4.5%, *p* < 0.001) of 1,702 tumors considered in a previous multi-center studies [5]. Several Japanese and Chinese studies found similar trends [6, 7]. 

The objective of this study was to confirm the disparity in the rate of benign histology among resected renal tumors suspected to be malignant based on imaging studies between Koreans and Americans, and to determine the potential racial effect of this phenomenon using current multi-institutional Korean-US data. The ultimate purpose of this study was to gain insights into reducing this type of hazardous overtreatment.

## Materials and methods

### Patient population and study design

This study was approved by the Institutional Review Board of Seoul National University Hospital (H-1608-034-783) in Korea and the US. We conducted a multi-institutional retrospective study. This study included nine tertiary, nationwide, academic hospitals in Korea and Johns Hopkins Hospital in the US. Although Johns Hopkins is a single institution, it is a large national referral hospital.

Patients who underwent resection of renal tumors due to suspected renal cell carcinoma (RCC) based on preoperative imaging were eligible for the study. Patients with clinical stage T1-4N0M0 were included, and patients with missing essential data were excluded from the study. We collected data on all possible cases of radical and partial nephrectomy, regardless of the final pathology, in consecutive series from each hospital between 1988 and 2015. In total, 11,529 patients (8,812 from Korea and 2,717 from the US) were included in the analysis.

### Study outcomes

The primary outcome was the rate of benign histology in the final pathology. We did not perform a central pathologic review because experienced pathologists will unlikely disagree on the distinction between malignant and benign renal tumors [8]. 

### Data collection and statistical analysis

We collected essential data on age at surgery, sex, race, pathology result (benign/malignancy), tumor size (largest pathologic diameter, if the unavailable size on computed tomography [9, 10] scan could be replaced), year of surgery, surgical method (open/laparoscopic/robotic), and type of surgery (partial/radical). Information on patients’ height, weight, body mass index (BMI), American Society of Anesthesiologists score, incidentaloma (yes/no), preoperative biopsy (yes/no), results of the previous biopsy (benign/malignancy/insufficient for diagnosis), preoperative clinical T stage, preoperative serum creatinine level, estimated glomerular filtration rate (eGFR), pathologic TNM stage, Fuhrman grade, and histologic subtype (RCC subtype and specific benign histology) were collected as the basic data set.

We compared the rates of benign histology in total and according to the tumor size (≤ 2.0, 2.1–4.0, 4.1–7.0, and > 7.0 cm) between Korean and American patients. Subgroup analyses were performed according to age (< 65 years and ≥ 65 years), sex, incidentaloma (yes/no), preoperative biopsy (yes/no), surgical method (open/laparoscopic/robotic), type of surgery (partial/radical), year of surgery (< 2000, 2001–2005, 2006–2010, and 2011–2015), and tumor size (≤ 2.0, 2.1–4.0, 4.1–7.0 and > 7.0 cm). Univariate and multivariable logistic regression analyses were performed for benign histology on the final pathology using clinically significant variables. Histological subtypes were also evaluated in these two countries. To evaluate the racial effect, we divided the patients into Korean, Asian in the US, and Non-Asian in the US, and compared the rates among the groups.

Propensity-score matching was performed to adjust powerful confounding factors. One-to-one matching without replacement was performed using the nearest-neighbor match on the logit of the propensity for three variables: age, sex, and tumor.

Multilevel logistic regression was performed to quantify the observed variation attributable to the institutional effect of clustering in the multilevel mixed-effect models. The median odds ratios (ORs) for random institutional effects were used to quantify the magnitude of the effect of clustering [11]. The median OR was used to quantify the increase in risk if one were to move from one institution to another institution with a higher risk for the benign histology [12]. 

For ad hoc analysis, we examined the correlation between the number of dedicated uro-radiologists in the hospital and the rate of benign histology among Korean institutions. We compared the rates using the chi-squared test and the significance level was set at *p* < 0.05.

## Results

The basic patient characteristics are summarized in Table 1. Korean patients were younger, more male-dominant, and had smaller tumors than those of US patients. The rate of benign histology among Korean patients was significantly lower than that among US patients (6.3% vs. 14.3%, *p* < 0.001). Table 2 shows the rates of benign histology according to the tumor size. The rates of benign histology in Korean patients were significantly lower than those of US patients for all size categories (*p* < 0.001), except for the tumor size > 7 cm. On correlation plot also, the linear correlation was prominent in tumors measuring 7 cm or less (Supplementary Fig. 1).

**Table 1:** Baseline characteristics of the patients

	Korea (*N* = 8,812)	United States (*N* = 2,717)	p
Age, year	55.3 ± 12.7	60.0 ± 12.1	< 0.001
Sex, No. (%)			< 0.001
Male	6,098 (69.2)	1,744 (64.2)	
Female	2,714 (30.8)	973 (35.8)	
BMI, kg/m^2^	24.5 ± 5.7	29.9 ± 6.8	< 0.001
Creatinine (mg/dL)	1.08 ± 1.22	1.22 ± 0.98	< 0.001
Preop. eGFR, mL/min/1.73m^2^	77.6 ± 33.8	68.2 ± 23.4	< 0.001
Race, No. (%)			< 0.001
Asian	8,812 (100)	69 (2.5)	
Caucasian	0	2125 (78.2)	
African-American	0	470 (17.3)	
Others	0	53 (2.0)	
Missing	0	0	
ASA score, No. (%)			< 0.001
I	3,363 (38.2)	20 (0.3)	
II	4,151 (47.1)	938 (44.5)	
III	352 (4.0)	1090 (51.7)	
IV	13 (0.1)	62 (2.9)	
Missing	933 (10.6)	0	
Clinical T stage, No. (%)			< 0.001
T1a	5,623 (65.8)	1,358 (50.2)	
T1b	1,489 (19.2)	711 (26.3)	
T2	696 (9.0)	362 (13.3)	
T3	294 (3.7)	173 (6.4)	
T4	25 (0.3)	9 (0.3)	
Missing	143 (1.8)	91 (3.4)	
Year of Surgery, No. (%)			< 0.001
− 2000	467 (6.0)	6 (0.2)	
2001–2005	781 (10.1)	606 (22.3)	
2006–2010	2,744 (35.4)	928 (34.2)	
2011–2015	3,757 (48.5)	1177 (43.3)	
Type of Surgery, No. (%)			0.639
Partial nephrectomy	4,761 (54.0)	1,454 (53.5)	
Radical nephrectomy	4,051 (46.0)	1,263 (46.5)	
Surgical Method, No. (%)			< 0.001
Open	3,893 (50.2)	540 (19.9)	
Laparoscopic	2,263 (29.2)	1341 (49.4)	
Robotic	1,593 (20.6)	836 (30.8)	
Tumor size, cm	4.0 ± 3.0	4.9 ± 3.2	< 0.001
Tumor Size, No. (%)			< 0.001
≤ 4 cm	5,295 (68.3)	1454 (53.6)	
> 4 cm, ≤ 7 cm	1,514 (19.5)	735 (27.1)	
> 7 cm	940 (12.1)	528 (19.4)	
Histology, No. (%)			< 0.001
Renal cell carcinoma	7145 (92.1)	2316 (85.2)	
Other malignancy	127 (1.6)	13 (0.5)	
Oncocytoma	121 (1.6)	197 (7.3)	
Angiomyolipoma	206 (2.7)	91 (3.3)	
Other benign tumors	150 (2.0)	100 (3.7)	

BMI, body mass index; ASA, American society of anesthesiologists; eGFR, estimated glomerular filtration rate

**Table 2:** Rate of benign histology of surgically-excised renal masses in the US and Korea

Size	Korea			United States			p
	No. of renal masses	No. of benign masses	Rate of benign histology	No. of renal masses	No. of benign masses	Rate of benign histology	
**≤ 2 cm**	2,405	243	**10.1%**	515	124	**24.1%**	< 0.001
**> 2 and ≤ 4 cm**	3,497	209	**6.0%**	939	160	**17.0%**	< 0.001
**> 4 and ≤ 7 cm**	1,780	60	**3.4%**	735	74	**10.1%**	< 0.001
**> 7 cm**	1,130	47	**4.2%**	528	30	**5.7%**	0.170
**Total**	8,812	559	**6.3%**	2,717	388	**14.3%**	< 0.001

Among benign tumors, angiomyolipomas were the most common (46.1%) in Korean patients, and oncocytomas were the most common (50.8%) in US patients. For further analysis, propensity score was matched (age, sex, and tumor size) and the result showed similar pattern as the entire cohort, showing significantly lower rates of benign histology in Korean patients (*p* < 0.001), except for the tumor size > 7 cm (Supplementary Tables 1 and Supplementary Table 2).

**Fig. 1:**
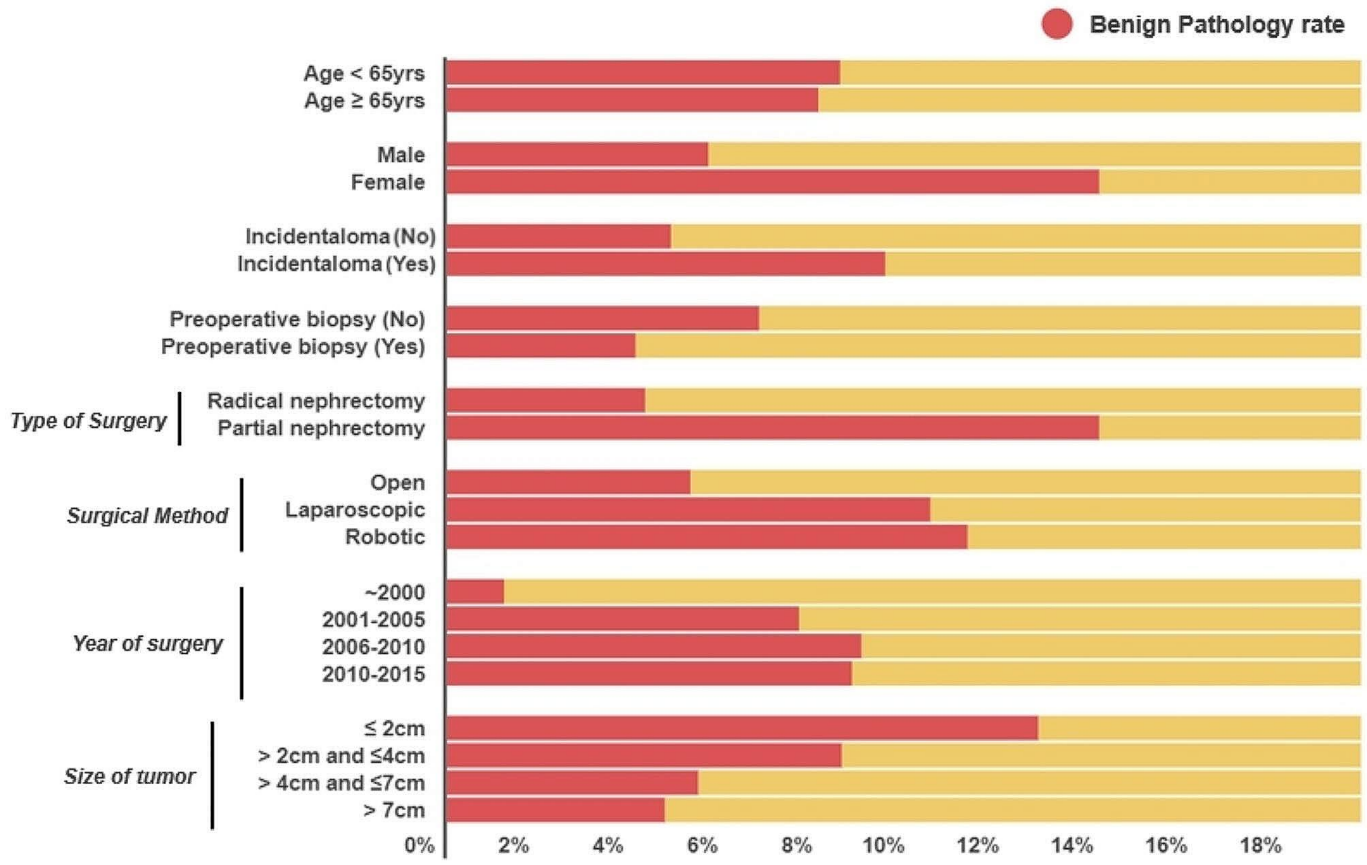
Stacked horizontal bar charts depicting benign histology rates according to the clinical categories shown in Table 3

Smaller tumor size, incidentaloma, partial nephrectomy, minimally invasive surgery (laparoscopic or robotic surgery), recent surgeries, and women were associated with a higher rate of benign histology than the others (Tables 3 and 4, and Fig. 1). After adjusting for other factors, the patient’s country was still a significant risk factor (Table 4). In the hierarchical regression analysis, there was a 10% variation between institutions in the rates of benign histology, as depicted by the interclass correlation. (Supplementary Table 3). The median OR was 1.76, indicating that a randomly selected patient at any given institution had 1.76-fold higher odds of being diagnosed with benign histology after surgery than an identical patient at a different random hospital.

**Table 3:** Subgroup analysis on rate of benign histology

Variables	Number of benign histology (%)	p
Age		0.459
< 65 years (*n* = 8,243)	687 (8.3)	
≥ 65 years (*n* = 3,285)	260 (7.9)	
Sex		< 0.001
Male (*n* = 7,842)	435 (5.5)	
Female (*n* = 3,687)	512 (13.9)	
Incidentaloma (Korea data only)		< 0.001
No (*n* = 4,862)	231 (4.8)	
Yes (*n* = 3,214)	300 (9.3)	
Preoperative biopsy (Korea data only)		0.165
No (*n* = 7,902)	524 (6.6)	
Yes (*n* = 175)	7 (4.0)	
Type of surgery		< 0.001
Radical (*n* = 5,314)	224 (4.2)	
Partial (*n* = 5,215)	723 (13.9)	
Surgical method		< 0.001
Open (*n* = 5,056)	262 (5.2)	
Laparoscopic (*n* = 4,043)	416 (10.3)	
Robotic (*n* = 2,430)	269 (11.1)	
Year of surgery		< 0.001
− 2000 (*n* = 486)	6 (1.2)	
2001–2005 (*n* = 1,550)	116 (7.5)	
2006–2010 (*n* = 3,975)	350 (8.8)	
2011–2015 (*n* = 5,518)	475 (8.6)	
Size of tumor		< 0.001
≤ 2 cm (*n* = 2,920)	367 (12.6)	
> 2 cm and ≤ 4 cm (*n* = 4,436)	369 (8.3)	
> 4 cm and ≤ 7 cm (*n* = 2,515)	134 (5.3)	
> 7 cm (*n* = 1,658)	77 (4.6)	

**Table 4:** Univariate and multivariable logistic regression analyses for benign histology on final pathology

Variables	Univariate			Multivariable		
	Odds ratio	95% CI	p-value	Odds ratio	95% CI	p-value
Country						
Korea	Reference			Reference		
United States	2.46	2.14–2.82	< 0.001	2.56	2.07–3.15	< 0.001
Age (years) (continuous)	0.99	0.99–1.00	0.020	0.99	0.98–0.99	< 0.001
Sex						
Male	Reference			Reference		
Female	2.75	2.40–3.14	< 0.001	2.62	2.24–3.05	< 0.001
Body mass index (kg/m^2^) (continuous)	1.01	1.00–1.02	0.192	0.98	0.96–0.99	0.003
Type of surgery						
Radical	Reference			Reference		
Partial	2.99	2.56–3.49	< 0.001	2.99	2.67–3.77	< 0.001
Surgical method			< 0.001			< 0.001
Open	Reference			Reference		
Laparoscopic	2.10	1.79–2.46	< 0.001	1.93	1.58–2.35	< 0.001
Robotic	2.28	1.91–2.72	< 0.001	1.33	1.08–1.65	0.008
Year of surgery			< 0.001			0.005
− 2000	Reference			Reference		
2001–2005	6.47	2.83–14.80	< 0.001	1.28	0.53–3.09	0.590
2006–2010	7.72	3.43–17.41	< 0.001	2.18	0.94–5.04	0.059
2010–2015	7.54	3.35–16.95	< 0.001	2.25	0.98–5.19	0.057
Size of tumor			< 0.001			< 0.001
≤ 2 cm	Reference			Reference		
> 2 cm and ≤ 4 cm	0.63	0.54–0.74	< 0.001	0.69	0.58–0.82	< 0.001
> 4 cm and ≤ 7 cm	0.39	0.32–0.48	< 0.001	0.61	0.47–0.79	< 0.001
> 7 cm	0.34	0.26–0.44	< 0.001	0.83	0.58–1.18	0.294

The rate of benign histology in patients who underwent preoperative biopsy did not differ from that in patients who did not (4.0% vs. 6.6%, *p* = 0.165) (Table 3; Fig. 1). Among patients who underwent preoperative biopsy (*n* = 175), 10 (5.7%) had non-diagnostic results, 128 (73.1%) had malignancy, and 37 (21.1%) had benign or favored a benign diagnosis based on biopsy pathology. However, 32 (86.5%, 32/37) patients had malignancy in their final pathology despite the benign biopsy results. The rate of benign histology in the non-diagnostic cases was 20% (2/10).

The rate of benign histology among SRMs in Asian patients in the US showed a higher tendency with borderline significance than that in Korean patients (15.8% (6/38) vs. 7.6% (451/5897), *p* = 0.061). It was similar to that in non-Asians in the US (15.8% vs. 19.6% (278/1415), *p* = 0.554).

**Fig. 2:**
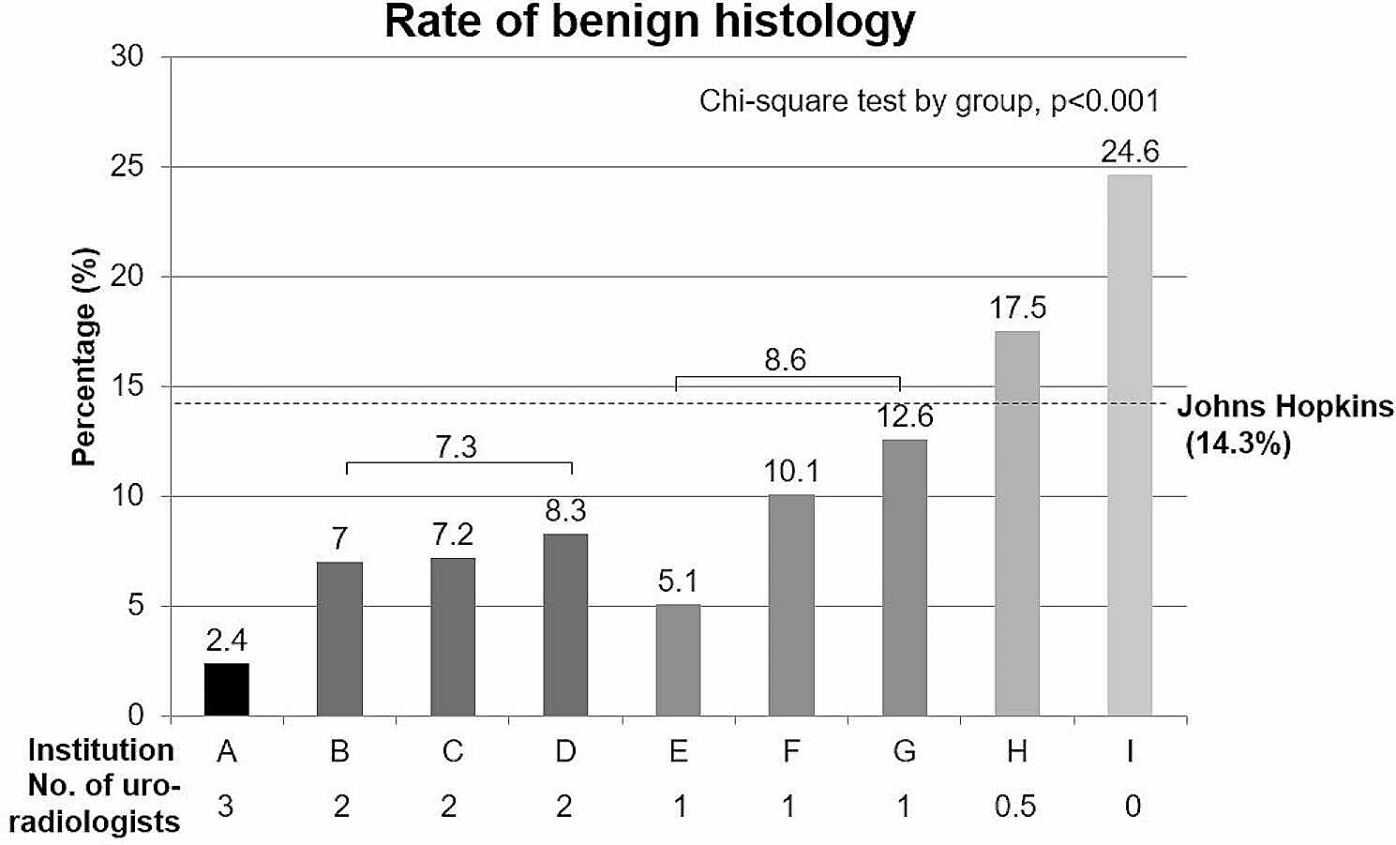
The rate of benign histology according to Korean institutions and number of dedicated uro-radiologist in each hospital

The rate of benign histology was inversely correlated with the number of dedicated uro-radiologists in Korean hospitals (*n* = 3; 2.4%, *n* = 2; 7.3%, *n* = 1; 8.6%, *n* = 0.5; 17.5%, and *n* = 0; 24.6%, *p* < 0.001) (Fig. 2). After adjusting for other factors (age, sex, and tumor size), the number of uro-radiologists was significant risk factor (Supplementary Table 4). The risk of benign histology decreased exponentially for each additional uro-radiologist.

## Discussion

The current US data in this study showed that similar rates of benign histology were reported in previous literatures [1]. ^, [10, 13]^ These rates were significantly higher than those in Koreans in all tumor size categories, except for tumors > 7 cm. Suppose the rate of benign histology among Asian patients in the US is similar to that of Koreans and significantly lower than that of non-Asian patients in the US, racial differences could be considered a major cause of this disparity. However, the rate of benign histology among Asians in the US was not different from that among non-Asians. Although the statistical power was limited because of the small number of Asian populations in the US, the benign histology rate was sufficiently higher than that in Koreans with borderline significance. Thus, we believe that differences in practice patterns and accessibility to medical services are major factors. Still, we should be very prudent for this conclusion for various environmental and modifiable risk factors. Urologists’ and patients’ attitudes regarding SRMs and the medicolegal situation may also contribute to the results. International data were demonstrated to be lower than those of the US in a previous meta-analyses [1]. Western countries, such as the Netherlands and Canada, have lower benign histology rates than the US and higher benign histology rates than Asian countries [13, 14]. However, there were wide variations among countries, and studies in each country.

The most likely explanation for this is the commitment of uro-radiologists to the diagnostic process. Most tertiary hospitals in Korea have assigned uro-radiologists, who mainly work in urologic clinics. They typically perform kidney ultrasonography (USG) by themselves and interpret kidney CT and magnetic resonance images (MRI). In contrast, professional sonographers may replace radiologists in many hospitals in the US. Furthermore, the interpretation of kidney images may not be performed solely by the assigned uro-radiologists in the US. There is no designated all-time uro-radiologist at Johns Hopkins Hospital also who participated in this study. Differentiation of SRMs as RCC or benign tumors in several types of medical images is a highly specialized area and requires specialized knowledge, which is challenging even for uro-radiologists [15]. Indeed, the number of dedicated uro-radiologists in a hospital was negatively correlated with the rate of benign histology after surgery in this study. It was observed that for each additional uro-radiologist, the benign histology rate decreases exponentially. This could be a reflection of case volume. Nevertheless, we believe manpower investment and uro-radiologist commitment might also be important in preventing this misdiagnosis. A higher BMI in the US may also contribute to a higher benign histology rate. This is because obesity leads to higher a signal-to-noise ratio, which may obscure subtle low-contrast lesions [16]. However, since the BMI of Asian patients in the US was similar to those of Koreans (25.3 ± 4.7 vs. 24.5 ± 5.7, *p* = 0.291), this may be partially attributable.

In our data, the most common benign histology was angiomyolipoma (46.1%) and oncocytoma (50.8%) in Korean and US patients, respectively. These findings are concordant with previous reports [5, 10, 17, 18]. Thus, additional sonography or MRI or machine learning based texture analysis should be considered in Korea to rule out angiomyolipoma when the characterization of a tumor is inconclusive. It can be diagnosed by a distinctive imaging pattern [15, 19]. In contrast, oncocytoma cannot be reliably distinguished from chromophobe RCC [18], and biopsy can be recommended when the tumor has oncocytic features in the US.

Some biopsy enthusiast groups advocate universal renal tumor biopsy (RTB) for all or most patients with SRMs to reduce unnecessary treatment based on diagnostic inaccuracy [20–22]. Even though there is high accuracy and a low risk of complications [20, 22, 23], we believe that unnecessary RTB is also another type of overtreatment. In our study, the utilization rate of RTB in Korea was only 2.0%. Nevertheless, the rate of benign histology in Korea was similar to that of centers that routinely perform RTB in Canada [24], and the rate of benign histology was similar in patients who underwent preoperative biopsy and in those who did not. A recent systematic review of RTB showed an overall non-diagnostic rate of 14.1% and a worrisome negative predictive value of 63.3%. Despite a negative biopsy result, the final pathology report of 36.7% of patients undergoing surgery revealed they had a malignant tumor [23]. In our study, this rate reached 86.5%. Thus, a more careful interpretation of imaging tests should be repeated if indicated, or alternative imaging tests should be given preference over RTB.

Some researchers prefer active surveillance of SRMs to reduce unnecessary surgery because their growth rates are slow and metastatic potential is very low [25–27]. However, most studies in the past dealt with watchful waiting, not real active surveillance for mostly old or surgically unfitted patients [27]. Prospective registries are now finding proper protocols and evidence; thus, active surveillance is not yet widely accepted [25, 28]. However, based on the experience of Seoul National University Hospital (SNUH), which showed the lowest rate of benign histology (2.4%) in this study, we carefully suggest that (1) actively monitoring tumor size growth rates [15, 25–27, 29], (2) alternating imaging tests (kidney protocol contrast CT [3 or 4 phasic], MRI, USG, and contrast USG for cystic mass [30], and 3) conducting and interpreting USG by uro-radiologist might be helpful.

In the meanwhile, recently, for the goal of distinguishing clear cell RCC from other renal tumors, including benign renal mass, Zirconium-89-girentuximab PET/CT, which targets CAIX membrane protein has been developed [31]. Phase III international clinical trial (NCT03849118) is now going on and expected to show promising result. When combined with the experience-based protocol of SNUH, there may be a synergistic effect in lowering the benign histology rate.

The limitations of this study should be addressed. A lack of a central pathological review may have caused some bias. However, this effect is minimal. We demonstrated difference of benign histology rate between Asian populations in the US and Koreans with borderline significance and attributed this as practice pattern and accessibility to medical services, however, we could not specifically consider the immigration generation, and their environmental and modifiable risk factors. The number of dedicated uro-radiologists may vary according to time. Nonetheless, the current number of assigned uro-radiologists may reflect the institutional experience and the amount of investment in it. The Korean sample did not represent the entire Asian population. Therefore, caution should be exercised when interpreting ethnic effects. Some patients in the US, particularly in earlier periods, may have undergone elective PN for well-distinguished angiomyolipoma. We could only evaluate some of the data regarding RTBs. We could analyze the performance results of the RTB for those who underwent surgery; thus, the results may be biased. We could not determine the rate of treatment avoidance due to RTB; however, we could calculate positive and negative predictive values among patients who underwent surgery, which were also clinically important data. The strength of this study is the relatively large number of patients (almost ten thousand, from representative institutions across Korea).

## Conclusions

We confirmed that the rate of benign histology among resected renal tumors in Korea was significantly lower than in the US. This disparity is not because of racial differences. It cannot be taken for granted that a substantial number of patients can be diagnosed with benign tumors after surgical removal. Further studies are warranted to significantly reduce overtreatment problems. Re-evaluation of the current standards of care is imperative for kidney tumors, which cannot be completely discounted as benign using an initial imaging test. Consequently, new protocols for better diagnosis must be prepared while avoiding both the extreme ends of overtreatment—the “treat-all” and “biopsy-all” paradigms.

### Electronic supplementary material

Below is the link to the electronic supplementary material.

Supplementary Material 1

Supplementary Material 2

Supplementary Material 3

Supplementary Material 4

Supplementary Material 5

## Data Availability

The datasets used and/or analysed during the current study are available from the corresponding author on reasonable request.

## Abbreviations

RCC: renal cell carcinoma
RTB: renal tumor biopsy
SRMs: small renal masses
PN: partial nephrectomy
