# Supplementary material for: Rate of benign histology after resection of suspected renal cell carcinoma: multicenter comparison between Korea and the United States
Source: BMC Cancer. 2024 Feb 15;24:216. doi: 10.1186/s12885-024-11941-3 (PMC10870474; doi:10.1186/s12885-024-11941-3)
Supplement: Supplementary file 1 — Supplementary Material 1 [file 12885_2024_11941_MOESM1_ESM.docx]

**Supplementary Table 1. Baseline characteristics of the propensity matched patients.**

|  | **Korea**  **(N=2,717)** | **United States (N=2,717)** | **p** |
| --- | --- | --- | --- |
| Age, year | 59.4 ± 12.7 | 60.0 ± 12.1 | 0.099 |
| Sex, No. (%) |  |  | 0.177 |
| Male | 1,696 (62.4) | 1,744 (64.2) |  |
| Female | 1,021 (37.6) | 973 (35.8) |  |
| Tumor size, cm | 5.1 ± 3.8 | 4.9 ± 3.2 | 0.108 |
| Histology, No. (%) |  |  | <0.001 |
| Renal cell carcinoma | 2505 (92.2) | 2316 (85.2) |  |
| Other malignancy | 49 (1.8) | 13 (0.5) |  |
| Oncocytoma | 37 (1.4) | 197 (7.3) |  |
| Angiomyolipoma | 73 (2.7) | 91 (3.3) |  |
| Other benign tumors | 49 (1.8) | 100 (3.7) |  |
