# Supplementary material for: Rate of benign histology after resection of suspected renal cell carcinoma: multicenter comparison between Korea and the United States
Source: BMC Cancer. 2024 Feb 15;24:216. doi: 10.1186/s12885-024-11941-3 (PMC10870474; doi:10.1186/s12885-024-11941-3)
Supplement: Supplementary file 2 — Supplementary Material 2 [file 12885_2024_11941_MOESM2_ESM.docx]

**Supplementary Table 2. Rate of benign histology of surgically-excised renal masses in the US and Korea in propensity score matched cohort (Age, Sex, Size matched)**

| **Size** | **Korea**  **(N=2,717)** | | | **United States**  **(N=2,717)** | | | **p** |
| --- | --- | --- | --- | --- | --- | --- | --- |
|  | **No. of renal masses** | **No. of benign masses** | **Rate of benign histology** | **No. of renal masses** | **No. of benign masses** | **Rate of benign histology** |  |
| **≤ 2 cm** | 615 | 65 | **10.6** | 515 | 124 | **24.1%** | <0.001 |
| **>2 and ≤4 cm** | 822 | 45 | **5.5%** | 939 | 160 | **17.0%** | <0.001 |
| **>4 and ≤7cm** | 617 | 23 | **3.7%** | 735 | 74 | **10.1%** | <0.001 |
| **>7cm** | 663 | 30 | **4.5%** | 528 | 30 | **5.7%** | 0.364 |
| **Total** | 2717 | 163 | **6.0%** | 2,717 | 388 | **14.3%** | <0.001 |
