# Supplementary material for: Rate of benign histology after resection of suspected renal cell carcinoma: multicenter comparison between Korea and the United States
Source: BMC Cancer. 2024 Feb 15;24:216. doi: 10.1186/s12885-024-11941-3 (PMC10870474; doi:10.1186/s12885-024-11941-3)
Supplement: Supplementary file 3 — Supplementary Material 3 [file 12885_2024_11941_MOESM3_ESM.docx]

**Supplementary Table 3.** Multilevel logistic regression analyses for benign histology on final pathology in 10 institutions

| **Variables** |  |  | **Multilevel logistic regression analysis** | | |
| --- | --- | --- | --- | --- | --- |
|  |  |  | **Odds ratio** | **95% CI** | **p-value** |
| Age (years) (continuous) |  |  | 0.99 | 0.98 – 0.99 | <0.001 |
| Sex |  |  |  |  |  |
| Male |  |  | Reference |  |  |
| Female |  |  | 2.66 | 2.27 – 3.10 | <0.001 |
| Body mass index (kg/m^2^) (continuous) |  |  | 0.98 | 0.96 – 0.99 | 0.005 |
| Type of surgery |  |  |  |  |  |
| Radical |  |  | Reference |  |  |
| Partial |  |  | 2.70 | 2.13 – 3.44 | <0.001 |
| Surgical method |  |  |  |  |  |
| Open |  |  | Reference |  |  |
| Laparoscopic |  |  | 1.19 | 0.94– 1.50 | 0.140 |
| Robotic |  |  | 1.11 | 0.88 – 1.40 | 0.388 |
| Year of surgery |  |  |  |  | 0.005 |
| - 2000 |  |  | Reference |  |  |
| 2001-2005 |  |  | 0.85 | 0.35 – 2.09 | 0.729 |
| 2006-2010 |  |  | 1.36 | 0.58 – 3.19 | 0.477 |
| 2010-2015 |  |  | 1.33 | 0.57 – 3.12 | 0.506 |
| Size of tumor |  |  |  |  | <0.001 |
| ≤ 2cm |  |  | Reference |  |  |
| > 2cm and ≤4cm |  |  | 0.68 | 0.57 – 0.81 | <0.001 |
| > 4cm and ≤7cm |  |  | 0.60 | 0.46 – 0.79 | <0.001 |
| > 7cm |  |  | 0.77 | 0.53 – 1.10 | 0.154 |
| **Random-effects of institutional variable** | | | | | |
| Variance of random effect |  |  | 0.35 |  |  |
| Interclass correlation |  |  | 0.10 |  |  |
| Median odds ratio |  |  | 1.76 |  |  |
