# Supplementary material for: Rate of benign histology after resection of suspected renal cell carcinoma: multicenter comparison between Korea and the United States
Source: BMC Cancer. 2024 Feb 15;24:216. doi: 10.1186/s12885-024-11941-3 (PMC10870474; doi:10.1186/s12885-024-11941-3)
Supplement: Supplementary file 4 — Supplementary Material 4 [file 12885_2024_11941_MOESM4_ESM.docx]

**Supplementary Table 4. Multivariable logistic regression analyses for benign histology on final pathology in terms of the number of uro-radiologist**

| **Variables** |  | **Multivariable** | | |
| --- | --- | --- | --- | --- |
|  |  | **Odds ratio** | **95% CI** | **p-value** |
| Country |  |  |  |  |
| Age (years) (continuous) |  | 0.98 | 0.97 – 0.99 | <0.001 |
| Sex |  |  |  |  |
| Male |  | Reference |  |  |
| Female |  | 3.17 | 2.65 – 3.78 | <0.001 |
| Size of tumor |  | 0.92 | 0.88-0.95 | <0.001 |
| Uro-radiologist number |  |  |  |  |
| Uro-radiologist No. : 0 |  | Reference |  |  |
| Uro-radiologist No. : 0.5 |  | 0.67 | 0.40-1.13 | 0.134 |
| Uro-radiologist No. : 1 |  | 0.42 | 0.27-0.66 | <0.001 |
| Uro-radiologist No. : 2 |  | 0.24 | 0.15-0.36 | <0.001 |
| Uro-radiologist No. : 3 |  | 0.08 | 0.05-0.14 | <0.001 |
