# Supplementary figures and images for: Rate of benign histology after resection of suspected renal cell carcinoma: multicenter comparison between Korea and the United States
Source: BMC Cancer. 2024 Feb 15;24:216. doi: 10.1186/s12885-024-11941-3 (PMC10870474; doi:10.1186/s12885-024-11941-3)

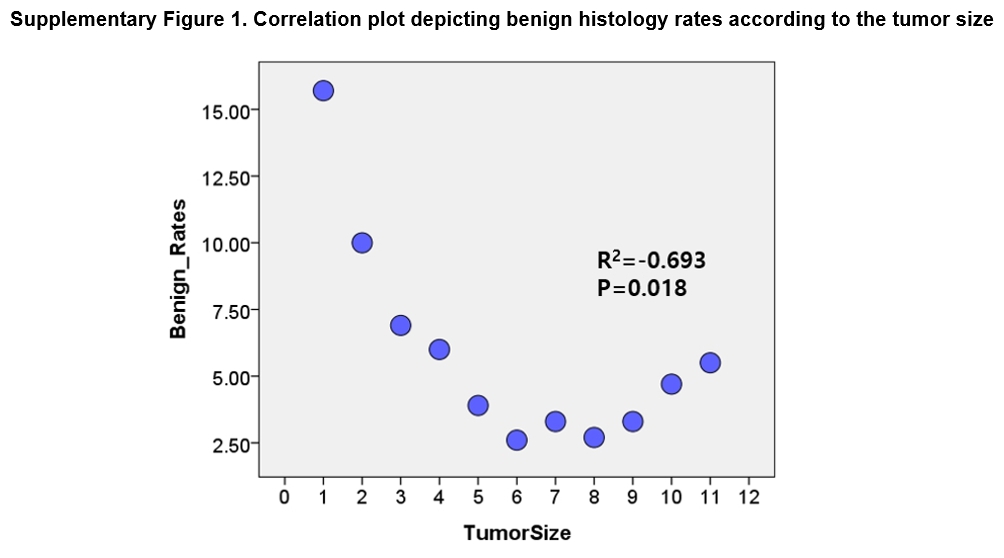

Supplement: Supplementary file 5 — Supplementary Material 5 [file 12885_2024_11941_MOESM5_ESM.jpg]
